# Primary intracranial sarcomas in Peruvian children: a high-incidence clinicopathological series from a National Reference Center

**DOI:** 10.3332/ecancer.2026.2133

**Published:** 2026-05-27

**Authors:** Sandro Casavilca-Zambrano, Arie Perry, Jenny Bonifacio-Mundaca, Luis Ojeda-Medina, Juan Luis García-León, Raúl Mantilla, Mauricio Ramal-Matute, Karin Castro-Aguirre, Rosdali Díaz-Coronado, Stéphane Bertani

**Affiliations:** 1 Faculty of Health Sciences, University of Huanuco, Huanuco 10001, Peru; 2 Department of Pathology, National Institute of Neoplastic Diseases (INEN), Lima 15038, Peru; 3 International Joint Laboratory of Molecular Anthropological Oncology (LOAM), IRD–INEN, Lima 15038, Peru; 4 Department of Pathology and Neurological Surgery, University of California, San Francisco, CA 94143, USA; 5 National Tumor Bank, Department of Pathology, National Institute of Neoplastic Diseases (INEN), Lima 15038, Peru; 6 Department of Neurosurgery, National Institute of Neoplastic Diseases (INEN), Lima 15038, Peru; 7 Department of Pediatric Oncology, Clinica Angloamericana y Clinica Delgado, Lima 15073, Peru; 8 Department of Research, National Institute of Neoplastic Diseases (INEN), Lima 15038, Peru; 9 Department of Pediatric Oncology, National Institute of Neoplastic Diseases (INEN), Lima 15038, Peru; 10 Faculty of Medicine, Cayetano Heredia Peruvian University (UPCH), Lima 15102, Peru; 11 UMR 152 PHARMADEV, IRD, University of Toulouse, Toulouse 31062, France; a https://orcid.org/0000-0002-0798-7544

**Keywords:** sarcoma, central nervous system neoplasms, DICER1 protein, ATRX protein, human, developing countries

## Abstract

**Background/Objectives:**

Primary intracranial sarcoma is a recognized entity within the World Health Organization Classification of Tumours of the Central Nervous System (CNS) and comprises an aggressive subgroup of mesenchymal, non-meningothelial neoplasms. Among these, tumours associated with DICER1 alterations have recently been characterized as a distinct molecular subset. Although globally rare, primary intracranial sarcomas consistent with the DICER1-associated spectrum demonstrate an unusually high incidence in Peru, where they represent the second most common high-grade pediatric CNS malignancy after medulloblastoma. This study aimed to describe their clinicopathological features and outcomes in a large national cohort.

**Methods:**

We retrospectively analyzed 112 pediatric cases diagnosed between 2011 and 2025 at the National Cancer Institute of Peru, integrating histopathology, immunohistochemistry and clinical outcomes.

**Results:**

The median age was 7 years, and most tumours were supratentorial, predominantly affecting the frontal and parietal lobes. Histologically, they exhibited spindle, pleomorphic and undifferentiated round cell patterns, with frequent hyaline globules and aberrant vasculature. Immunohistochemistry showed recurrent ATRX loss, p53 overexpression and high proliferative indices, whereas myogenic markers were variably expressed. Median overall survival was 25 months, with 12-, 36- and 60-month survival rates of 65.7%, 45.9% and 44.0%, respectively. None of the tested markers (ATRX, p53 and Ki-67) demonstrated prognostic significance.

**Conclusion:**

This is the largest pediatric series of DICER1-mutant primary intracranial sarcomas reported from a low- and middle-income country. The findings confirm their poor prognosis and biological heterogeneity, highlighting the urgent need for integrated molecular and epidemiological research to identify risk factors and improve patient outcomes.

## Introduction

The 2021 World Health Organization (WHO) classification of central nervous system (CNS) tumours introduced the category ‘tumours of uncertain differentiation’, which comprises (FUS (fused in sarcoma)‑EWSR1 (Ewing sarcoma breakpoint region 1)‑TAF15 (TAF15 RNA polymerase II transcription factor) family proteins and CREB (cAMP response element‑binding protein))]fusion–positive intracranial mesenchymal tumours, Capicua transcriptional repressor-rearranged sarcomas and primary intracranial sarcomas with DICER1 (PIS-DICER1) [1]. These mesenchymal, non-meningothelial tumours are thought to arise from progenitor cells within the meningeal coverings of the brain and along the perivascular Virchow–Robin spaces [2–4]. As mesoderm-derived structures, they retain the potential to generate tumours of multiple lineages, with DICER1 playing a crucial role in embryonic development, cell migration and proliferation [5–7].

Although exceedingly rare, CNS sarcomas represent a diagnostic and therapeutic challenge. Globally, they account for less than 0.5% of soft tissue sarcomas, which constitute only 1% of all malignancies [1,2,8]. Strikingly, in Peru, their reported incidence reaches 0.19 cases per 100,000 children, making them the second most common high-grade pediatric CNS malignancy after medulloblastoma [9,10].

In 2018, our group reported the first international cohort of 22 intracranial sarcomas harboring DICER1 mutations, establishing these tumours as a distinct molecular entity [11]. That study demonstrated frequent co-alterations in the mitogen-activated protein kinase pathway (including KRAS and NF1 mutations or deletions), often in combination with TP53 mutations and, in some cases, MYC or MYCN amplifications. Although most cases occur in children, adult and older adult patients were also affected [12]. This work contributed to the recognition of DICER1-mutant sarcomas as a distinctive subset, later incorporated into the 2021 WHO classification of CNS tumours. However, that study did not address regional epidemiology or provide data from low- and middle-income countries (LMICs), where incidence and biology may differ.

More recently, we have encountered tumours with melanocytic differentiation, raising the differential diagnosis of melanocytic meningeal and melanocytic nerve sheath tumours. Beyond the CNS, DICER1-related neoplasms include pleuropulmonary blastoma, cystic nephroma, Sertoli-Leydig cell tumour and ovarian rhabdomyosarcoma [12]. Notably, in our series, these mutations were also detected in tumours not previously linked to hereditary DICER1 syndrome [13].

Despite these advances, data on DICER1-mutant primary intracranial sarcomas from LMICs remain scarce, even though their incidence appears unusually elevated in Peru. To address this knowledge gap, we present here the largest pediatric cohort reported to date, integrating comprehensive clinicopathologic and molecular analyses to further define the spectrum of these rare and aggressive tumours.

## Materials and methods

### Study design and case selection

We retrospectively analyzed 112 pediatric patients with primary intracranial sarcoma diagnosed at the National Cancer Institute of Peru (INEN). Histological slides and formalin-fixed, paraffin-embedded (FFPE) tissue blocks from 2011 to 2025 were available for all cases, and clinical and demographic data were retrieved from institutional records.

### Molecular analysis

Molecular data were not generated within the scope of the present study. Instead, we incorporated previously published results from our earlier collaborative work [13], in which targeted next-generation sequencing (NGS) was performed on a subset of tumours using a custom capture panel for DICER1 and selected sarcoma-related genes. Of the 112 cases included in the current cohort, 10 had been analyzed in that prior study and were confirmed to harbor pathogenic DICER1 mutations.

The remaining cases did not undergo molecular testing and were included in the present study based on clinicopathologic criteria consistent with the previously molecularly validated DICER1-associated primary intracranial sarcoma entity described in the Peruvian population. Notably, earlier studies analyzing high-grade primary intracranial sarcomas from Peru demonstrated DICER1 alterations in all tested cases (40/40), along with a highly distinct DNA methylation profile, supporting the reproducibility of this tumour entity within our setting.

For the current analysis, previously published DICER1 mutational data were incorporated into clinicopathologic correlations. Cases labeled as ‘NR’ correspond to tumours with confirmed pathogenic DICER1 mutations for which specific variant classifications were not available in the original dataset.

### Histopathological evaluation

Histopathological evaluation was independently conducted by two expert oncologic pathologists (S.C.Z. and K.C.A.) specializing in CNS sarcomas. Tumours were classified based on predominant histology, with detailed documentation of features such as heterologous differentiation, vascular patterns and dissemination along perivascular Virchow–Robin spaces.

### Immunohistochemistry

Immunohistochemical staining of FFPE sections employed a panel of antibodies, including Desmin, MyoD1, CD99, Vimentin, S100, SOX10, CD56, ATRX, p53 and Ki-67. ATRX loss was defined as a complete absence of nuclear staining in tumour cells, with positive internal controls. Overexpression of p53, indicating potential *TP53* mutation, was identified by strong nuclear staining in ≥50% of tumour cells. The Ki-67 labeling index was calculated as the percentage of positively stained nuclei in proliferative areas (or ‘hot spots’).

### Statistical analysis

Descriptive statistical analyses summarized sociodemographic and clinical variables. Qualitative variables were reported as frequencies and percentages, while quantitative variables were described using measures of central tendency and dispersion: the mean with minimum and maximum values for variables with a symmetric or normal distribution, and the median with interquartile range (IQR; Q1–Q3) for variables with a skewed distribution.

Overall survival (OS), defined as the time from diagnosis to death or last follow-up, was estimated using the Kaplan–Meier method, with survival curves compared via the log-rank test. Patients lost to follow-up immediately after diagnosis were excluded from survival analysis.

The optimal Ki-67 cut-off for predicting mortality was determined by receiver operating characteristic (ROC) curve analysis. All analyses were conducted in R software (R Foundation for Statistical Computing, Vienna, Austria), with statistical significance set at *p* < 0.05.

### Ethics

This study complied with the Declaration of Helsinki and was approved by the INEN Institutional Review Board (INEN; approval: INEN 22-15). Given the retrospective nature of the study, the ethics committee waived informed consent requirements. Patient data were anonymized to safeguard confidentiality.

## Results

### Patient characteristics

The clinical and demographic characteristics of the 112 patients included in this study are summarized in Table 1. The median age at diagnosis was 7 years (IQR: 5–9), highlighting the predominantly pediatric nature of the cohort. There was a male predominance (61.6%). Lima was the most frequent region of birth (48.2%), consistent with its demographic predominance and the location of INEN as the national referral center. However, cases were identified from nearly all regions of the country, suggesting broad geographic distribution rather than a localized clustering. This observation may be relevant for future epidemiologic studies investigating potential environmental or population-related factors associated with the unusually high incidence reported in Peru.

The frontal lobe was the most common tumour location (40%), followed by the parietal lobe (23%) and the posterior fossa (14%) (Table 2).

The median follow-up time was 9 months (IQR: 4–40). At the last follow-up, 48 patients (42.9%) were alive, 41 (36.6%) had died and 23 (20.5%) were lost to follow-up.

### Histopathologic and immunohistochemical findings

**Figure 1.**
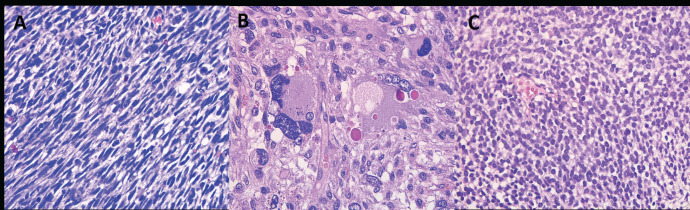
Representative histological and immunohistochemical patterns observed in primary intracranial sarcomas. (A) Spindle cell histologic pattern on hematoxylin and eosin (H&E) staining. Image at 20× objective magnification (total magnification 200×) demonstrates elongated, fusiform tumour cells arranged in intersecting fascicles, consistent with a spindle cell phenotype. (B) Pleomorphic histologic pattern on H&E staining. The section shows markedly pleomorphic tumour cells with irregular nuclear contours, prominent hyperchromasia and frequent multinucleation, arranged in a disorganized, sheet-like architecture, characteristic of high-grade pleomorphic sarcoma. (C) Undifferentiated round cell histologic pattern on H&E staining. Solid growth of small, round to oval primitive tumour cells with a high nuclear-to-cytoplasmic ratio, consistent with an undifferentiated round cell tumour.

The complete immunohistochemical profile is presented in Table 3, and representative histologic and immunohistochemical images are shown in Figures 1 and 2. Tumours were classified into three predominant histologic patterns: spindle cell, pleomorphic and undifferentiated round cell (Figure 1).

Histological examination revealed additional morphologic features that varied in frequency and distribution (Figure 2), including hyaline globules, eosinophilic granular intracytoplasmic inclusions, myxoid stroma, focal xanthomatous change, diffuse cytoplasmic granularity, epithelioid morphology and scattered ballooned tumour cells. In a subset of cases, heterologous differentiation was present, most commonly myogenic differentiation and rhabdoid morphology and less frequently with chondroid or melanocytic components.

A meningoangiomatous dissemination pattern was a frequent finding, characterized by tumour extension along Virchow–Robin spaces and infiltration into adjacent dura or cerebral parenchyma. Abnormal vascular architecture was also observed, including markedly dilated intratumoural vessels, angiomatoid formations and hemangiopericytoid patterns (Figure 2).

Reticulin silver staining demonstrated consistent pericellular reticulin fiber deposition, ranging from delicate to dense. Immunohistochemistry showed Desmin positivity in 43 of 82 cases (52.4%), MyoD1 expression in 19 of 68 cases (27.9%) and CD99 expression in 11 of 17 tested cases (64.7%). S100 was positive in seven of 41 cases (17.1%), SOX10 in three of 16 (18.7%) and CD56 in 11 of 12 (91.7%). Overexpression of p53 (≥50% nuclear staining) was observed in 60 of 71 cases (84.5%) and loss of nuclear ATRX expression in 35 of 64 (54.7%). The median Ki-67 proliferation index was 70% (IQR: 50%–80%).

### Molecular findings

Molecular findings were not generated within the scope of the present study. Instead, we incorporated previously published targeted NGS results from our earlier series [13], which included ten of the tumours analyzed here. In that study, DICER1 mutations were identified in all cases (*n* = 10). Four tumours harbored pathogenic variants for which the specific amino acid change was not available (classified as NR). Among cases with defined variants, recurrent missense substitutions in the RNase IIIb domain included D1709N (*n* = 2), E1705K (*n* = 4) and G1809R (*n* = 1). These residues are critical for metal ion binding and cleavage of the 5 p arm of precursor microRNAs [14]; substitutions at these sites impair miRNA processing and contribute to oncogenesis in DICER1-associated sarcomas [15].

Nonsense mutations introducing premature stop codons – Q1028X, W400X and R1003X – were also reported, likely resulting in truncated proteins with loss of catalytic activity. Some tumours harbored combined alterations, most commonly Q1028X with E1705K and R1003X with D1709N, consistent with the ‘hotspot + loss-of-function’ pattern previously described in high-grade DICER1-mutant sarcomas [16]. This combination suggests biallelic inactivation through a catalytic domain hotspot mutation paired with a truncating variant, a mechanism associated with aggressive clinical behaviour.

No germline DICER1 testing was performed in that cohort, and none of the patients had clinical features suggestive of the hereditary DICER1 tumour predisposition syndrome. These data, retrieved from the earlier study, are summarized in Table 4.

### Overall survival

**Figure 3.**
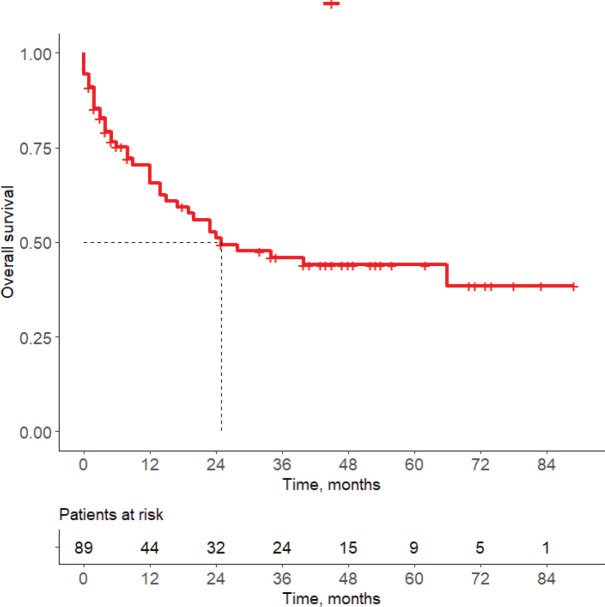
Kaplan–Meier estimate of OS.

**Figure 4.**
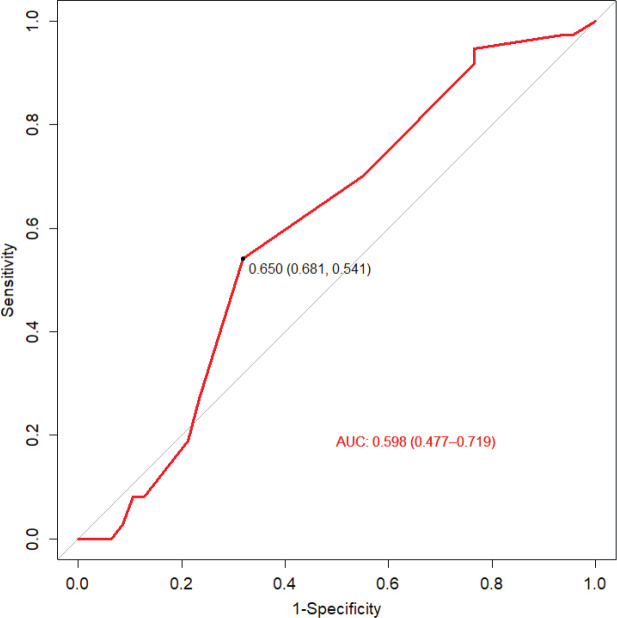
ROC curve for optimal Ki-67 cutoff in mortality.

Among patients with available follow-up data (*n* = 89), 41 deaths (46.1%) were recorded. The median OS was 25 months, with OS rates at 12, 36 and 60 months of 65.7%, 45.9% and 44.0%, respectively (Figure 3). The optimal cut-off value for Ki-67 expression for mortality prediction was 65% (*n* = 101), yielding an area under the curve (AUC) of 0.598 (95% CI: 0.477–0.719), with a sensitivity of 54.1% and specificity of 68.1% (Figure 4). The AUC did not differ significantly from 0.5, indicating limited predictive value. Neither ATRX loss nor p53 overexpression showed statistically significant prognostic impact in multivariate analysis.

## Discussion

In Peru, CNS tumours represent the second most common group of pediatric malignancies, following leukemia [10,17]. Within this category, primary intracranial sarcomas – particularly those consistent with the DICER1-associated spectrum – remain rare but clinically relevant. Despite limited access to advanced molecular platforms in many LMICs, accurate diagnosis can be achieved through an integrated approach combining histomorphology, targeted immunohistochemistry and clinicoradiologic correlation [13].

Recent genomic studies have refined the taxonomy of DICER1-associated mesenchymal tumours into three categories: (1) low-grade mesenchymal tumours with DICER1 alteration, typically indolent; (2) sarcomas with DICER1 alteration ; and (3) PIS-DICER1, which behave aggressively and frequently harbor co-mutations in TP53, KRAS/NRAS, KMT2D and NF1 [18]. Pathogenic variants in the RNase IIIb domain of DICER1 impair precursor miRNA processing, leading to dysregulated gene expression and oncogenesis in mesenchymal neoplasms [6,7,18]. In our series, molecular data were derived from previously published collaborative work confirming DICER1 mutations in a subset of cases. Importantly, earlier molecular analyses of Peruvian pediatric high-grade primary intracranial sarcomas demonstrated DICER1 alterations in all tested tumours (40/40), establishing a reproducible molecular entity within our population. In the present cohort, molecular confirmation was available in ten cases. Limited access to comprehensive molecular testing represents an inherent challenge in our LMIC setting [9, 13].

The median OS was 25 months (95% CI: (15–NA)). Survival rates at 12, 36 and 60 months were 65.7%, 45.9% and 44.0%, respectively. Censored observations are indicated by tick marks. The table below the graph shows the number of patients at risk at each time point.

The histopathologic spectrum of our cohort included spindle cell, pleomorphic and undifferentiated round cell patterns, in line with prior descriptions [2]. Additional features such as hyaline globules, eosinophilic cytoplasmic inclusions, heterologous differentiation and meningoangiomatous dissemination further support alignment with previously characterized DICER1-associated sarcomas [19]. A distinctive and frequent finding was meningoangiomatous dissemination, characterized by infiltration along Virchow–Robin spaces and occasional extension into dura or brain parenchyma.

Our findings are broadly consistent with published literature, while also revealing distinct locoregional patterns. Earlier reports described a predominance of parietal and temporal tumours [3, 20], whereas our cohort demonstrated frontal and frontoparietal predominance. Prior work from our group led by Diaz Coronado *et al* [9] systematically described frequent intratumoural hemorrhage and vascular malformations in Peruvian cases. In the present study, neuroimaging was not systematically reviewed for prognostic correlation, and therefore, the potential survival impact of these features could not be assessed. Prospective studies integrating standardized radiologic, molecular and clinical data are warranted.

The optimal cut-off for Ki-67 expression was 65%, with an AUC of 0.598 (95% CI: 0.477–0.719), sensitivity of 54.1% and specificity of 68.1%. The AUC did not differ significantly from 0.5, indicating limited predictive value

Molecular markers of prognostic relevance remain uncertain. ATRX loss was frequent but not associated with survival, differing from observations in soft tissue sarcomas [19, 21]. Similarly, p53 overexpression and Ki-67 index did not demonstrate independent prognostic significance. These findings highlight the complexity of risk stratification in primary intracranial sarcomas.

From a therapeutic perspective, management at our institution follows a standardized algorithm established by the INEN, Peru (RJ-N° 258-2024-J-INEN). Treatment is based on maximal safe resection followed by risk-adapted multimodal therapy, including Ifosfamide, Carboplatin, Etoposide-based chemotherapy and focal radiotherapy when indicated. Despite aggressive management, survival outcomes remain limited, underscoring the need for improved stratification and collaborative international efforts [22].

Although comprehensive molecular testing was not available for all cases, prior molecular characterization of Peruvian pediatric intracranial sarcomas demonstrated consistent DICER1 alterations, supporting the clinicopathologic framework applied in this study.

In summary, our findings demonstrate that PIS-DICER1-associated spectrum in Peruvian children share key features with international series, including aggressive behaviour, high proliferative activity and supratentorial predominance. At the same time, we identify distinctive characteristics within our population, such as frontal predominance and frequent vascular abnormalities. As the largest pediatric cohort reported to date from an LMIC, this study provides important insight into a geographically enriched tumour entity and highlights the need for continued global collaboration to optimize diagnostic and therapeutic strategies.

## Conclusion

The diagnosis of pediatric CNS sarcomas remains a significant challenge, particularly in LMICs where molecular platforms are often limited. Although focal myogenic marker expression may be observed, PIS-DICER1 should not be misclassified as rhabdomyosarcoma. These tumours encompass a heterogeneous histological spectrum, frequently combining spindle cell, pleomorphic and undifferentiated round cell components, and may occasionally exhibit heterologous differentiation.

In our series, ATRX loss, p53 overexpression and high proliferative index did not demonstrate prognostic significance, underscoring the lack of reliable biomarkers for risk stratification. The unusually high incidence of these tumours in Peru further highlights the need to integrate molecular profiling with epidemiological and environmental studies to investigate potential contributory factors.

Moving forward, multidisciplinary collaboration and international data sharing will be essential to refine diagnostic criteria, identify prognostic markers and develop targeted therapies for this rare but aggressive pediatric tumour type.

## Conflicts of interest

The authors declare no conflicts of interest.

## Funding

This research received no external funding.

## Institutional review board

The study was conducted in accordance with the Declaration of Helsinki and approved by the Institutional Review Board of the Instituto Nacional de Enfermedades Neoplásicas, Peru (project approval number: INEN 22-15).

## Data availability

The authors confirm that the data supporting the findings of this study are available within the article.

## Author contributions

Conceptualization, methodology, validation, formal analysis, investigation, resources, data curation, writing – original draft preparation, writing – review and editing, visualization, supervision, project administration and funding acquisition. All authors have read and agreed to the published version of the manuscript.

## Figures and Tables

**Figure 2.**
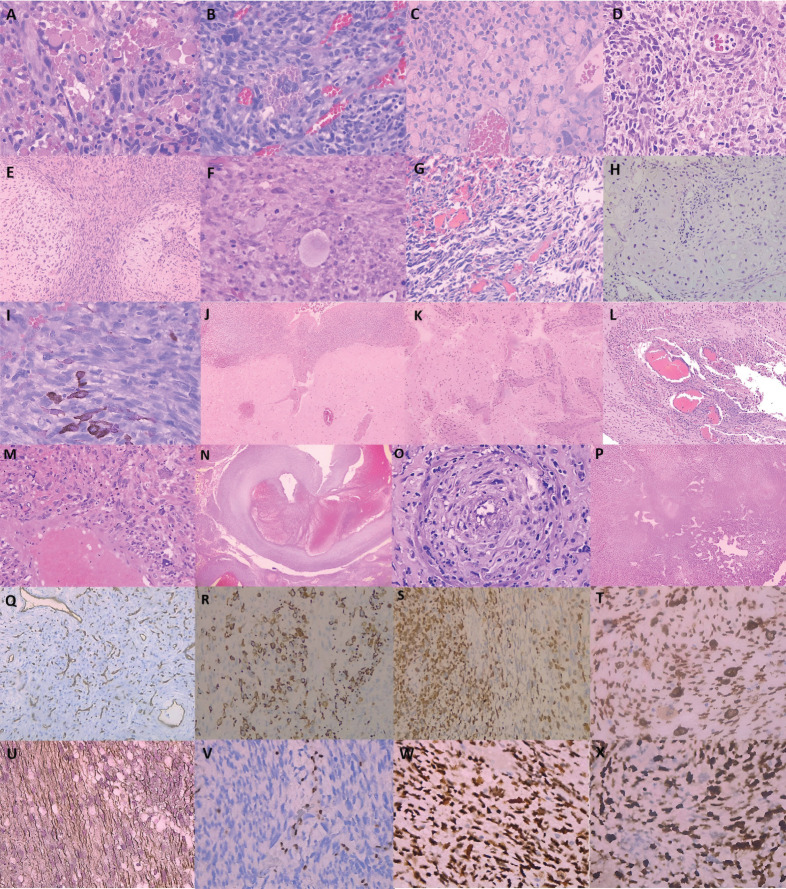
Representative histological and immunohistochemical patterns observed in primary intracranial sarcomas. (A) Hyaline globules on hematoxylin and eosin (H&E) staining, 20× objective (total magnification 200×). (B) Multinucleated pleomorphic tumour cells with marked nuclear hyperchromasia and acidophilic intracytoplasmic deposits (H&E, 20×). (C) Xanthomatous clear cell change with a solid growth pattern (H&E, 20×; total magnification 200×). (D) Diffuse cytoplasmic granularity. (E) Epithelioid features. (F) Scattered ballooned cells (all H&E, 20×). (G) Heterologous differentiation with spindle-shaped rhabdoid cells in variably extensive myxoid stroma. (H) Chondroid areas and, less frequently, (I) melanocytic components (H&E, 20×). (J) Widespread meningoangiomatous dissemination. (K) Tumour involvement of perivascular Virchow–Robin spaces (H&E, 20×). (L) Extension into adjacent dura mater (H&E, 5×; total magnification 50×). (M) Angiomatoid features (H&E, 10×). (N) Irregular vascular architecture with markedly dilated intratumoural vessels, suggestive of aberrant angiogenesis (H&E, 5×). (O) Neoplastic infiltration of vascular walls (H&E, 10×). (P) Hemangiopericytoid vascular pattern (H&E, 5×). (Q) Endothelial CD34 positivity highlighting the vascular architecture (CD34 immunostaining, 10×). (R) Desmin and (S) MyoD1 immunostaining showing variable myogenic differentiation, from focal to extensive. (T) TLE1 with diffuse, strong nuclear reactivity (20×; total magnification 200×). (U) Reticulin silver stain demonstrating a pericellular reticulin network, ranging from delicate to dense, outlining the stromal framework (20×). (V) Loss of nuclear ATRX expression. (W) Diffuse strong nuclear p53 positivity. (X) Markedly elevated Ki-67 proliferative index, frequently >40% and occasionally approaching 100% (all at 20×).

**Figure 2.**
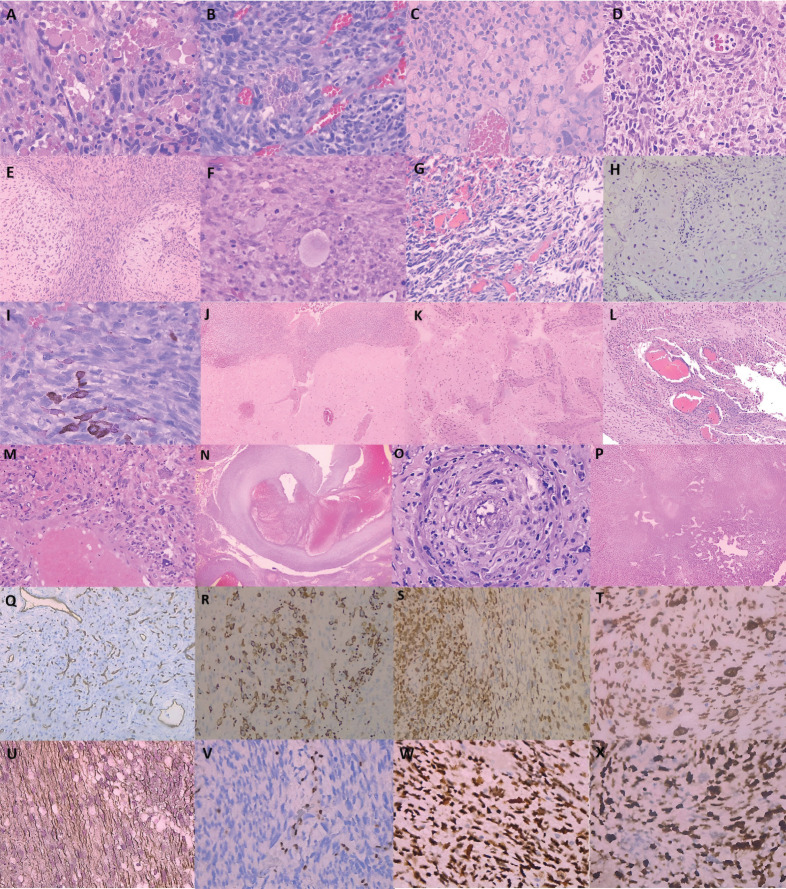
Representative histological and immunohistochemical patterns observed in primary intracranial sarcomas. (A) Hyaline globules on hematoxylin and eosin (H&E) staining, 20× objective (total magnification 200×). (B) Multinucleated pleomorphic tumour cells with marked nuclear hyperchromasia and acidophilic intracytoplasmic deposits (H&E, 20×). (C) Xanthomatous clear cell change with a solid growth pattern (H&E, 20×; total magnification 200×). (D) Diffuse cytoplasmic granularity. (E) Epithelioid features. (F) Scattered ballooned cells (all H&E, 20×). (G) Heterologous differentiation with spindle-shaped rhabdoid cells in variably extensive myxoid stroma. (H) Chondroid areas and, less frequently, (I) melanocytic components (H&E, 20×). (J) Widespread meningoangiomatous dissemination. (K) Tumour involvement of perivascular Virchow–Robin spaces (H&E, 20×). (L) Extension into adjacent dura mater (H&E, 5×; total magnification 50×). (M) Angiomatoid features (H&E, 10×). (N) Irregular vascular architecture with markedly dilated intratumoural vessels, suggestive of aberrant angiogenesis (H&E, 5×). (O) Neoplastic infiltration of vascular walls (H&E, 10×). (P) Hemangiopericytoid vascular pattern (H&E, 5×). (Q) Endothelial CD34 positivity highlighting the vascular architecture (CD34 immunostaining, 10×). (R) Desmin and (S) MyoD1 immunostaining showing variable myogenic differentiation, from focal to extensive. (T) TLE1 with diffuse, strong nuclear reactivity (20×; total magnification 200×). (U) Reticulin silver stain demonstrating a pericellular reticulin network, ranging from delicate to dense, outlining the stromal framework (20×). (V) Loss of nuclear ATRX expression. (W) Diffuse strong nuclear p53 positivity. (X) Markedly elevated Ki-67 proliferative index, frequently >40% and occasionally approaching 100% (all at 20×).

**Table 1.** Clinical and demographic characteristics of pediatric primary intracranial sarcomas (n = 112).

Characteristic	Value
Age, years, median (IQR)	7 (5–9)
Sex	
Male	69 (61.6%)
Female	43 (38.4%)
Region of birth	
Lima	54 (48.2%)
Cajamarca	6 (5.4%)
Piura	5 (4.5%)
Junín	4 (3.6%)
Cusco	3 (2.7%)
Ancash	2 (1.8%)
Apurímac	2 (1.8%)
Arequipa	2 (1.8%)
Huancavelica	2 (1.8%)
La Libertad	2 (1.8%)
Lambayeque	2 (1.8%)
Amazonas	1 (0.9%)
Callao	1 (0.9%)
Ica	1 (0.9%)
Pasco	1 (0.9%)
Tacna	1 (0.9%)
Not recorded	23 (20.5%)
Follow-up, months, median (IQR)	9 (4–40)
Survival status	
Deceased (event)	41 (36.6%)
Alive (censored)	48 (42.9%)
Lost to follow-up (censored)	23 (20.5%)

IQR: interquartile range; Censored: includes patients alive at last follow-up and those lost to follow-up

**Table 2.** Anatomical location of tumours.

Anatomical site	n (%)
Frontal lobe	45 (40.0)
Parietal lobe	26 (23.0)
Posterior fossa	16 (14.0)
Other sites	25 (22.0)

Locations are based on the dominant site of tumour involvement as determined by preoperative neuroimaging and/or intraoperative findings

**Table 3.** Immunohistochemical profile of primary intracranial sarcomas.

Marker	n	Negative n (%)	Positive n (%)	Remarks
Desmin	82	39 (47.6%)	43 (52.4%)	—
MyoD1	68	49 (72.0%)	19 (28.0%)	—
CD99	17	6 (35.3%)	11 (64.7%)	—
WT1	2	2 (100.0%)	0 (0.0%)	Only 2 cases
Vimentin	20	1 (5.0%)	19 (95.0%)	—
S100	41	34 (82.9%)	7 (17.1%)	—
p53	71	11 (15.5%)	60 (84.5%)	—
ATRX	64	35 (54.7%)	29 (45.3%)	—
SOX10	16	13 (81.3%)	3 (18.7%)	—
CD56	12	1 (8.3%)	11 (91.7%)	—
Ki-67	—	—	—	Median 70% (IQR 50%–80%)

IQR: interquartile range; Percentages calculated over tested cases for each marker. Positive: any unequivocal staining above background in the appropriate cellular compartment. Negative: complete absence of staining. Median Ki-67 labeling index is reported with IQR

**Table 4.** Summary of DICER1 mutations detected in analyzed cases.

Mutation type	Variant(s)	n
Missense	D1709N, E1705K, G1809R	7
Nonsense	Q1028X, W400X, R1003X	3
Nonsense + missense	Q1028X + E1705K; W400X + E1705K; R1003X + D1709N	3
NR	—	4

NR: mutation detected; variant classification not available. Missense mutations are recurrently located in the RNase IIIb domain and affect metal ion–binding residues; nonsense mutations introduce premature stop codons. Combined alterations represent ‘hotspot + truncating’ patterns consistent with biallelic inactivation
